# The risk factors in diabetic foot ulcers and predictive value of prognosis of wound tissue vascular endothelium growth factor

**DOI:** 10.1038/s41598-024-64009-4

**Published:** 2024-06-19

**Authors:** Jing Xu, Jian Gao, Hui Li, Zhoujun Zhu, Junliang Liu, Chong Gao

**Affiliations:** 1 Department of Oncology, The Second People’s Hospital of Lianyungang, No. 41 Hailiandong Road, Haizhou District, Lianyungang, 222006 China; 2 Department of Orthopedics, The Sixth Affiliated Hospital of Xinjiang Medical University, No. 39 Wuxingnan Road, Tian Shan District, Urumqi, 830002 China; 3 Department of Internal Medicine, Urumqi Maternal and Child Health Care Hospital, No. 3838, Convention and Exhibition Avenue, Midong District, Urumqi, 831400 China; 4 Department of Orthopedics, Weihai Stomatological Hospital, No. 268, Tongyi South Road, Huancui District, Weihai, 264299 China; 5 Department of Orthopedics, The Second People’s Hospital of Lianyungang, No. 41 Hailiandong Road, Haizhou District, Lianyungang, 222006 China

**Keywords:** Diabetes mellitus, Diabetic foot ulcers, Vascular endothelium growth factor, Risk factors, Retrospective study, Endocrine system and metabolic diseases, Diabetes

## Abstract

Diabetic foot ulcer (DFU) is a leading cause of high-level amputation in DM patients, with a low wound healing rate and a high incidence of infection. Vascular endothelial growth factor (VEGF) plays an important role in diabetes mellitus (DM) related complications. This study aims to explore the VEGF expression and its predictive value for prognosis in DFU, in order to provide basis for the prevention of DFU related adverse events. We analyzed 502 patients, with 328 in healing group and 174 in non-healing/recurrent group. The general clinical data and laboratory indicators of patients were compared through Spearman correlation analysis, ROC analysis and logistic regression analysis. Finally, the independent risk factors for adverse prognosis in DFU patients were confirmed. Spearman analysis reveals a positive correlation between the DFU healing rate and ABI, VEGF in wound tissue, and positive rate of VEGF expression, and a negative correlation with DM duration, FPG, HbA1c, TC, Scr, BUN, and serum VEGF. Further logistic regression analysis finds that the DM duration, FPG, HbA1c, ABI, serum VEGF, VEGF in wound tissue, and positive rate of VEGF expression are the independent risk factors for adverse prognosis in DFU (*p* < 0.05). DM duration, FPG, HbA1c, ABI, serum VEGF, VEGF in wound tissue, and positive rate of VEGF expression are the independent risk factors for prognosis in DFU patients. Patients with these risk factors should be screened in time, which is of great significance to prevent DFU related adverse events and improve outcomes.

## Introduction

Diabetic foot (DF) is a common complication of diabetes mellitus (DM) patients, which is clinically manifested as foot infection, ulcer or deep tissue destruction, with an alarming incidence rate of 20%. It is long term incurable, and even leads to amputation^1^. Epidemiological data show that the amputation rate of DF patients is 19.03%, while the annual mortality rate is as high as 16.26%, which seriously threatens the life quality and safety of patients^2^. Peripheral vascular disease is the main cause of diabetic foot ulcer (DFU). When the blood vessels are ischemic, it is necessary for lower extremity to establish the collateral circulation through arteriogenesis and angiogenesis. During this period, the role of vascular growth factor is particularly important^3^. As the main angiogenesis factor, vascular endothelial growth factor (VEGF) is involved in cancer, infection and other vascular dependent diseases. Current studies have confirmed that the level of serum VEGF in DM patients is associated with DM related complications, especially the increase in the severity of lower extremity vascular disease. There are various isoforms of VEGF. VEGF-A promotes early events of angiogenesis, including endothelial cell migration and proliferation. VEGF-A can improve the re-epithelization of diabetes wounds, and can be applied to the treatment of chronic wounds such as DFU, venous stasis ulcer and pressure ulcer with local skin ischemia. VEGF-C accelerates diabetic wound healing by enhancing angiogenesis as well as lymphangiogenesis in the granulation tissue. Generation of new lymphatic vessels in the wound should facilitate the exit of excess fluid and leukocytes and concomitantly decrease the edema associated with the inflammatory response. Also VEGF-A possesses lymphangiogenic effects but these effects were not as pronounced as with VEGF-C^4–6^. However, there are few clinical studies on the correlation between expression of VEGF in serum/tissue and wound healing in DFU patients with chronic refractory wounds^7^.

Therefore, analyzing the expression of VEGF in wound tissue has important clinical significance for the prevention and treatment of DFU. This study aims to explore the VEGF expression and its predictive value for prognosis in different degrees of DFU, in order to provide basis for clinicians to prevent DFU related adverse events.

## Methods

### Patient case selection

We obtained ethical approval exemption from the Second People’s Hospital of Lianyungang Ethics Committee to perform this study since we did not have direct contact with the participants. Informed consent was obtained from all participants and/or their legal guardians. We confirm that all methods were performed in accordance with the relevant guidelines and regulations. Patients treated for Type 2 DM combined with DFU in database records of our hospital were retrospectively analyzed from Jan 1, 2017, to Mar 31, 2023 (updated medical record system was used since Jan 1, 2017). Those who had complete medical records and met the 1999 WHO diabetes diagnostic criteria^8^ were selected. The exclusion criteria including: (1) patients diagnosed as Type 1 or gestational DM; (2) patients with acute complications such as diabetic ketoacidosis and hyperosmolar coma; (3) patients with primary liver and kidney function impairment, and/or blood and immune system diseases; and (4) patients receiving glucocorticoid, Immunosuppressive drug or chemotherapy drugs. The healing of DFU was defined as: (1) complete wound healing within one year of treatment, with no new ulcers; (2) wound not healed within one year of treatment; (3) original ulcer healed within one year of treatment, new ulcer at the healed ulcer site; and (4) original ulcer healed within one year of treatment, new ulcer in other areas or on the other foot. According to Wagner’s grading, there were 35 cases of level 1, 247 cases of level 2, 139 cases of level 3, 61 cases of level 4 and 20 cases of level 5.

Based on whether the wound healed after treatment, all patients were divided into a healing group and an non-healing/recurrent group.

### Observation indicators

The general clinical data were collected respectively, including age, sex, DM duration and ankle brachial index (ABI, the ratio of systolic pressure between ankle artery and brachial artery). Meanwhile, the laboratory indicators of patients were compared, including fasting plasma glucose (FPG), total cholesterol (TC), triglyceride (TG), low-density lipoprotein cholesterol (LDL-C), high-density lipoprotein cholesterol (HDL-C), blood urea nitrogen (BUN), C-reactive protein (CRP), serum creatinine (Scr), serum uric acid (SUA), glycosylated hemoglobin (HbA1c). All the above indicators were derived from the patient database.

In addition, the expression of VEGF was measured by the authors using ELISA (in serum) and Western Blot (in wound tissue). The peripheral venous blood (1.5 ml) was collected and then centrifuged at 3000 r for 10 min to separate red blood cells. Blood anticoagulant was added and the blood was centrifuged again at 3000 r for 10 min. 100 μl of supernatant was taken for testing. The absorbance of the sample was tested at a wavelength of 450 nm by ELISA reader. The wound tissues from both groups were collected and placed in 1.5 ml tubes. Protein was extracted by adding lysis solution, and VEGF was determined after electrophoresis, electroporation, elution, blocking, overnight incubation with mouse-anti human VEGF antibody, elution, secondary antibody, and then re-elution. The remaining tissue was fixed with 4% 0.01 mol/L (pH = 7.4) polyformaldehyde, dehydrated with ethanol, made transparent with xylene, and embedded in paraffin for section. Rabbit-anti human monoclonal VEGF antibody was added (1:100), incubated in water bath for 1 h, and then secondary antibody for 30 min. After immunohistochemical staining, the uniformly stained area was observed under a high-power microscope.

### Statistical analysis

Statistical analysis was conducted using Statistical Packages of Social Sciences (SPSS) software (version 26.0). Quantitative data following a normal distribution were represented as $\overline{x} \pm s$, and comparisons between two groups were made using the *t*-test. Meanwhile, nonnormally distributed variables was compared through non parametric tests. Categorical data were represented as *n* (%), and analyzed using the chi-square test. The correlation between DFU and various indicators was analyzed using Spearman correlation analysis. The factors which had close correlation with poor healing of DFU were further analyzed using logistic regression analysis. We used the receiver operating characteristic (ROC) curve to evaluate the non-healing risk of wounds in DFU which was predicted by VEGF expression. Statistical significance was set at *p* < 0.01 or *p* < 0.05.

### Ethics approval and consent to participate

The study was reviewed by the Second People’s Hospital of Lianyungang Ethics Committee. We obtained ethical approval exemption since we did not have direct contact with the participants.

## Result

A total of 502 consecutive cases are involved in the current study, including 235 males and 267 females with a mean age of 65.71 ± 9.43 years (ranges from 38 to 79 years). There are 328 cases in the healing group and 174 cases in the non-healing/recurrent group. The detailed patient information is shown in Table 1.

**Table 1:** Comparison of general data and laboratory indicators between the two groups.

Group	Number (male/female)	Age (years)	DM duration (years)	ABI	FPG (mmol/L)	TC (mmol/L)
Non-healing/recurrent	174 (85/89)	66.21 ± 8.75	9.84 ± 3.02	0.59 ± 0.20	8.97 ± 3.54	6.45 ± 1.08
Healing	328 (150/178)	65.44 ± 9.93	6.07 ± 1.15*	0.82 ± 0.26*	8.03 ± 2.11*	4.36 ± 0.81*

Group	TG (mmol/L)	LDL-C (mmol/L)	HDL-C (mmol/L)	HbA1c (%)	CRP (mmol/L)	BUN (mmol/L)
Non-healing/recurrent	1.31 ± 0.24	2.16 ± 0.39	1.17 ± 0.30	10.76 ± 3.12	9.38 ± 3.14	8.26 ± 4.52
Healing	1.25 ± 0.19	2.98 ± 0.72*	1.54 ± 0.43*	7.51 ± 2.48*	4.76 ± 2.25*	5.10 ± 2.45*

Group	SUA (μmol/L)	Scr (μmol/L)	VEGF in plasma (μg/L)	VEGF in wound tissue (μg/L)	Positive rate of VEGF expression (n, %)	
Non-healing/recurrent	319.74 ± 92.16	84.51 ± 29.76	1.83 ± 0.14	1.28 ± 0.05	116 (66.67)	
Healing	358.05 ± 87.22*	62.47 ± 23.59*	1.49 ± 0.20*	1.61 ± 0.08*	282 (85.98)*	

*The difference between the two groups is significant.

Diabetes mellitus (DM), ankle brachial index (ABI), glucose (FPG), total cholesterol (TC), triglyceride (TG), low-density lipoprotein cholesterol (LDL-C), high-density lipoprotein cholesterol (HDL-C), glycosylated hemoglobin (HbA1c), C-reactive protein (CRP), blood urea nitrogen (BUN), serum uric acid (SUA), serum creatinine (Scr), vascular endothelial growth factor (VEGF).

### Comparative analysis of general data and laboratory indicators

In the non-healing/recurrent group, the DM duration, FPG, TC, HbA1c, CRP, BUN, Scr, and serum VEGF are significantly higher than those in the healing group (*p* < 0.001, *p* = 0.035, *p* < 0.001,* p* = 0.017,* p* < 0.001, *p* = 0.009, *p* = 0.012, *p* = 0.026, respectively). Conversely, ABI, LDL-C, HDL-C, SUA, VEGF in wound tissue, and positive rate of VEGF expression are significantly lower than those in the healing group (*p* < 0.001, *p* = 0.019, *p* = 0.030, *p* = 0.013,* p* = 0.024, *p* = 0.008, respectively) (Table 1).

### Spearman correlation analysis between DFU and various indicators

The Spearman correlation analysis shows a positive correlation between the healing rate of DFU and ABI, HDL-C, LDL-C, VEGF in wound tissue, and positive rate of VEGF expression (*p* = 0.008, *p* = 0.018,* p* = 0.032,* p* < 0.001, *p* < 0.001, respectively). A negative correlation is observed with the DM duration, FPG, HbA1c, TC, Scr, BUN, and serum VEGF (*p* < 0.001, *p* = 0.020,* p* = 0.019,* p* = 0.017,* p* = 0.025,* p* = 0.015, *p* < 0.001, respectively), as shown in Table 2.

**Table 2:** Spearman correlation analysis between the healing rate of DFU and various indicators.

Item	DM duration	ABI	FPG	HbA1c	TC	Scr
Healing rate						
* r*	− 0.921	0.806	− 0.572	− 0.649	− 0.195	− 0.163
* p*	< 0.001	0.008	0.020	0.019	0.017	0.025

Item	BUN	LDL-C	HDL-C	VEGF in plasma	VEGF in wound tissue	Positive rate of VEGF expression
Healing rate						
* r*	− 0.124	− 0.107	0.131	− 0.812	0.705	0.958
* p*	0.015	0.032	0.018	< 0.001	< 0.001	< 0.001

Diabetes mellitus (DM), ankle brachial index (ABI), glucose (FPG), glycosylated hemoglobin (HbA1c), total cholesterol (TC), serum creatinine (Scr), blood urea nitrogen (BUN), low-density lipoprotein cholesterol (LDL-C), high-density lipoprotein cholesterol (HDL-C), vascular endothelial growth factor (VEGF).

### ROC analysis of VEGF and its predictive value for prognosis

**Figure 1:**
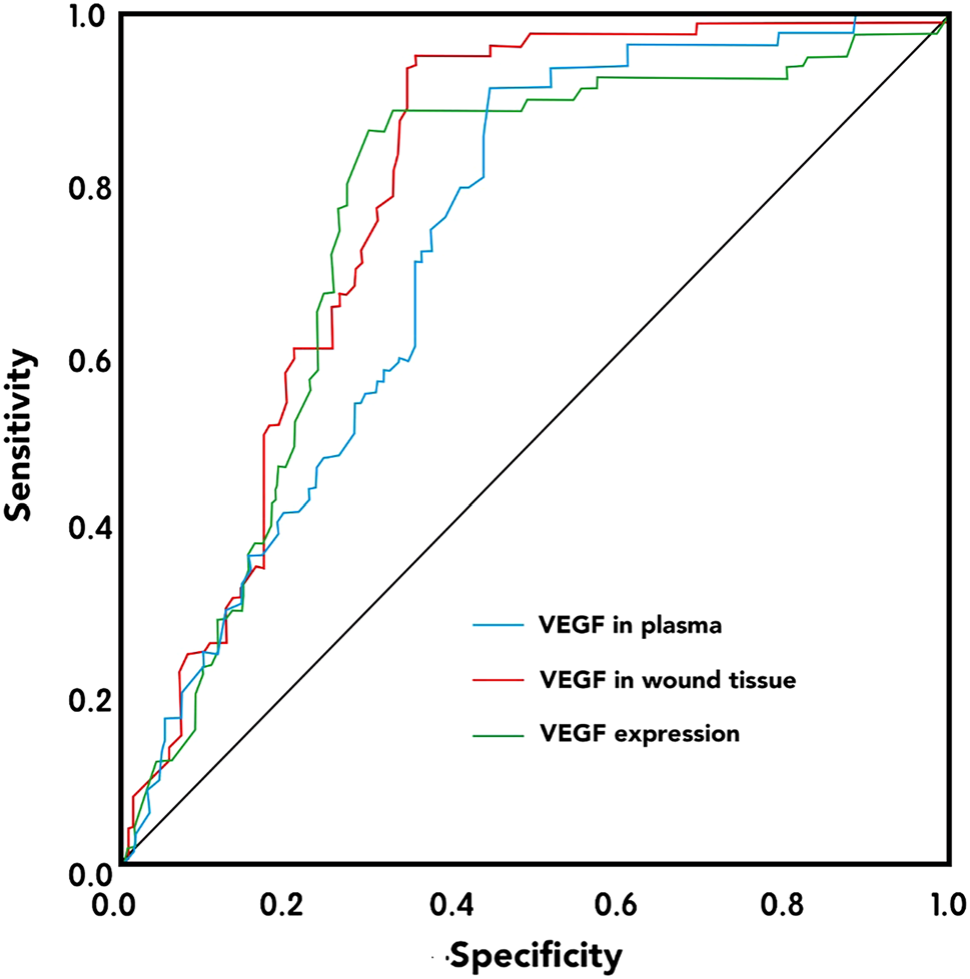
ROC curve for predicting wound healing in patients with DFU.

The area under the ROC curve (AUC) for serum VEGF is 0.728 (*p* < 0.001), with a cut-off point of 1.77 μg/L, a sensitivity of 89.50%, and a specificity of 57.30%. The AUC for VEGF in wound tissue is 0.790 (*p* < 0.001), with a cut-off point of 1.24 μg/L, a sensitivity of 93.00%, and a specificity of 65.10%. The AUC for positive rate of VEGF expression in wound tissue is 0.759 (*p* < 0.001), with a sensitivity of 89.10%, and a specificity of 74.30%. These results suggest that the level of VEGF and its expression in the local ulcer wounds have predictive value for the long-term prognosis of patients (Fig. 1).

### Logistic regression analysis of risk factors for DFU

The logistic regression analysis is conducted with DFU as the dependent variable and DM duration, ABI, FPG, HbA1c, TC, Scr, BUN, HDL-C, LDL-C, serum VEGF, VEGF in wound tissue, and positive rate of VEGF expression as the independent variables. The analysis shows that DM duration, ABI, FPG, HbA1c, serum VEGF, VEGF in wound tissue, and positive rate of VEGF expression are significant risk factors for non-healing/recurrent DFU (*p* < 0.001,* p* = 0.013,* p* < 0.001,* p* = 0.020,* p* < 0.001,* p* < 0.001,* p* < 0.001, respectively) (Table 3).

**Table 3:** Logistic regression analysis of risk factors for non-healing/recurrent DFU.

Variable	β	SE	Waldχ^2^	OR (95% CI)	p
DM duration	0.973	0.216	20.292	2.646 (1.047–4.329)	< 0.001*
ABI	− 0.468	0.205	5.212	0.626 (0.368–0.943)	0.013*
FPG	1.137	0.340	11.183	3.117 (1.382–5.295)	< 0.001*
HbA1c	0.612	0.285	4.611	1.844 (1.047–4.616)	0.020*
TC	0.146	0.124	1.386	1.157 (0.986–1.408)	0.185
Scr	0.282	0.419	0.453	1.326 (0.675–2.132)	0.359
BUN	0.091	0.075	1.472	1.095 (0.791–1.405)	0.117
HDL-C	− 0.505	0.683	0.547	0.604 (0.228–1.237)	0.281
LDL-C	− 1.069	0.732	2.133	0.343 (0.015–1.076)	0.094
Serum VEGF	0.106	0.028	14.332	1.112 (1.008–1.317)	< 0.001*
VEGF in wound tissue	− 1.385	0.224	38.230	0.250 (0.074–0.765)	< 0.001*
Positive rate of VEGF expression	− 0.824	0.161	26.194	0.439 (0.156–0.824)	< 0.001*

*The factor has statistical significance to non-healing/recurrent DFU.

Diabetic foot ulcer (DFU), diabetes mellitus (DM), ankle brachial index (ABI), glucose (FPG), glycosylated hemoglobin (HbA1c), total cholesterol (TC), serum creatinine (Scr), blood urea nitrogen (BUN), high-density lipoprotein cholesterol (HDL-C), low-density lipoprotein cholesterol (LDL-C), vascular endothelial growth factor (VEGF).

### Model discrimination and consistency validation

The discriminatory ability of the model is assessed using the concordance index (C-index), yielding a calculated C-index of 0.896 (95% CI 0.782–0.997, *p* < 0.01). This result indicates a strong discriminatory power of the model in predicting the risk of poor wound healing in DFU patients. The goodness-of-fit test for the predictive model revealed no significant discrepancy between the predicted and actual values (*p* > 0.05), suggesting that the model is robust and aligns well with the actual probability of risk occurrence, thereby demonstrating high calibration and reliability.

## Discussion

Studies have indicated that a hyperglycemic environment predisposes to distal limb neuropathy, vascular disease, and peripheral circulatory disorders^9,10^. This results in a lack of normal neuroprotective mechanisms in the foot, reduced blood supply, inadequate nutrient provision, and ultimately, local ulceration or even total foot necrosis. DFU is a leading cause of high-level amputation in DM patients, with a low wound healing rate and a high incidence of infection. The 5-year postoperative survival rate is less than 60%, significantly impacting patients' physical and mental health and quality of life^11^.

In this study, the Spearman analysis reveals a positive correlation between the DFU healing rate and ABI, VEGF in wound tissue, and positive rate of VEGF expression, and a negative correlation with DM duration, FPG, HbA1c, TC, Scr, BUN, and serum VEGF. However, after further multivariate logistic regression analysis, it is found that the DM duration, FPG, HbA1c, ABI, serum VEGF, VEGF in wound tissue, and positive rate of VEGF expression are the independent risk factors for adverse prognosis in DFU patients.

Long-term increase in blood glucose level can inhibit cell growth, promote vascular endothelial apoptosis, and induce arteriosclerosis, which is detrimental to the healing of the wound^12^. The content of HbA1c depends on the concentration of blood glucose and the duration of contact between blood glucose and hemoglobin. It is not affected by such factors as the time of blood collection, fasting and insulin use. Compared with blood glucose measurement, it has fewer influencing factors and can better reflect the long-term control of blood glucose level^13^. In addition, a longer DM duration usually brings more pain to patients, making them less compliant to the treatment, which means that it is more difficult for patients to control the blood glucose level^14^. This study suggests that when the DM duration, FPG and HbA1c are higher, non-healing or recurrence is more likely to occur. However, the meta-analysis of Huang et al.^15^ shows that there is no significant relationship between the DM duration and the recurrence of DFU. The regional and ethnic differences may be the reason for the contrary.

ABI is a screening and diagnostic tool for peripheral artery disease recommended by the American Diabetes Association. The decrease of blood flow is an important reason for the recurrence of DFU, and ABI is an important indicator used to evaluate vascular status. Previous studies have also shown that ABI is closely related to the Wagner grading of DF^16,17^. The abnormal ABI indicates arterial ischemia or calcification which leads to vascular endothelial cell damage and variation, peripheral neuropathy, and even extremity gangrene^18^. The result of this study suggests that DFU are more prone to adverse prognosis when ABI is lower, which is consistent with most studies^12,19^.

VEGF is a key regulator of vascular function, with vascular endothelial cells serving as both the target and source of VEGF^20^. In the context of injury, VEGF stimulates endothelial cell mitosis, enhances vascular permeability, and protects neurons, thereby promoting angiogenesis. Microcirculatory disturbance is one of the important pathophysiological bases of DM associated peripheral neuropathy. Poor blood glucose control in DM patients can lead to vascular structural changes such as thickening of microvascular basement membrane, swelling and proliferation of endothelial cells, and increased vascular permeability. These changes in microcirculation can cause the occurrence and development of DM associated peripheral neuropathy by causing energy metabolism disorder, sorbitol accumulation and inositol depletion, and abnormal increase of oxidative stress. At this time, the level of VEGF increases to promote angiogenesis and neural repair. Our results indicate that DFU patients in non-healing/recurrent group show higher serum VEGF. Therefore, higher serum VEGF is associated with DM associated peripheral neuropathy which is a precursor for DFU. The expression of VEGF in wound tissue is influenced by local oxygen concentration and hormone levels, and it synergizes with other cytokines and growth factors to facilitate wound repair^21^. Meanwhile, VEGF can also indirectly increase collateral circulation flow and restore blood supply. Studies have found that it can promote the repair of skin wound in DFU rats by regulating the increase of VEGF level, and the VEGF expression level in skeletal muscle of DFU patients is significantly lower than that of non-DM patients with lower extremity atherosclerosis and occlusion^22,23^. At all time points after injury, the VEGF expression level on wound surface of DM rats is lower than that of normal rats. Moreover, the decrease in VEGF expression level in ischemic extremities will lead to a significant reduction in collateral circulation around the occluded vessels^24^. Finally, the low VEGF expression level causes insufficient collateral compensation and progression of refractory persistent ischemic ulcers. Our result indicates that the VEGF expression has important clinical significance in judging prognosis of DFU. In view of this, VEGF may be a therapeutic target for DFU. Given the escalating incidence of DM, understanding the role of VEGF expression in DFU wound tissue is crucial for patient prognosis, wound healing, prevention of secondary infections, and effective management of DFU recurrence.

The present study, to our knowledge, is the first study evaluating related risk factors and simultaneously exploring VEGF expression in wound tissues and its predictive value of prognosis in DFU. However, there are still several limitations in the current study, Firstly, the relatively small sample size (because of our newly updated medical record system). Expanding our sample population will better eliminate data bias and reduce errors as much as possible, or even obtain a definite subversive result. Secondly, microvascular complications (diabetic retinopathy, diabetic nephropathy, etc.) were not included in our analysis as potential risk factors because of limited evaluable data. Gazzaruso et al.^25^ showed that the absence of diabetic retinopathy and nephropathy were independently associated with DFU healing, and retinopathy was a predictor both of minor amputation and mortality. Moreover, a 7-year follow-up cohort study by Olesen et al.^26^ indicated that microvascular disease was independently associated with a threefold increase of amputation risk. These findings imply that microvascular complications of DM may be used as potential markers of worse outcomes in DF. Thirdly, this study did not include TcPO2 as an evaluating indicator of DM peripheral vascular disease. We should note that ABI may lose its diagnostic power when artery calcifications presents. Literature has showed that TcPO2 is a potential predictor of major adverse cardiovascular events among patients with uncomplicated type 2 DM and that its predictive value seems to be greater than that of ABI^27^. Finally, the subjects included in this study all belong to single center, which relatively limiting the research region. Therefore, high-quality, large sample, and multicenter clinical and basic mechanism studies should be performed in our future clinical work to provide physicians with the best evidence-based information.

## Conclusion

In conclusion, DM duration, FPG, HbA1c, ABI, serum VEGF, VEGF in wound tissue, and positive rate of VEGF expression are the independent risk factors for prognosis in DFU patients. Patients with these risk factors should be screened in time, which is of great significance to prevent DFU related adverse events and improve outcomes. In addition, these risk factors may be considered as predictors for adverse prognosis of DFU, providing evidence for the prevention and treatment of severe complication such as gangrene requiring amputation in future works.

## Data Availability

All supporting data can be provided upon request to the authors. Jing Xu should be contacted if someone wants to request the data.
